# Laparoscopic Management of Small Bowel Obstruction and Ischemia Caused by Spontaneous Transomental Hernia in a Virgin Abdomen: A Case Report

**DOI:** 10.1155/cris/6668120

**Published:** 2026-04-29

**Authors:** Alaa Mousli

**Affiliations:** ^1^ Department of Surgery, Faculty of Medicine, King Abdulaziz University, Jeddah, Makkah, Saudi Arabia, kau.edu.sa

## Abstract

Transomental hernias (TOHs) are a rare form of internal hernia, accounting for ~1%–4% of all internal hernias and 0.5%–3% of bowel obstructions. Preoperatively diagnosing TOH is challenging owing to nonspecific obstructive symptoms and the absence of a hernia sac. This challenge can lead to delayed interventions and increased risk of bowel ischemia. In this report, we present the case of a 71‐year‐old female patient with a negative surgical history who presented with small bowel obstruction and a high lactate level. Abdominal computed tomography (CT) showed a closed‐loop obstruction with wall thickening and decreased enhancement, raising concerns of ischemia. Laparoscopic exploration revealed spontaneous TOH through the greater omentum, resulting in small bowel obstruction and ischemia, which were successfully managed entirely with laparoscopic resection, anastomosis, and closure of the mesenteric defect. The patient’s postoperative course was uneventful. This report discusses diagnostic challenges, surgical approaches, and key intraoperative findings.

## 1. Introduction

Internal hernias occur when the viscera protrude through a peritoneal or mesenteric defect while remaining within the abdominal cavity. Transomental hernias (TOHs) are a rare subtype in which the abdominal viscera, commonly the small bowel, protrude through a defect in the greater or lesser omentum. TOH can occur postoperatively, following trauma, after peritoneal inflammation, or, rarely, as in this case, spontaneously in patients with a negative surgical history and absence of previous trauma. Small bowel obstruction is a common presentation. Owing to its potential for strangulation, TOH is associated with a high postoperative mortality rate of ~30% [1, 2], which emphasizes the importance of early diagnosis and prompt surgical management [3, 4]. This report discusses diagnostic challenges, surgical approaches, and key intraoperative findings in a patient with small bowel obstruction and ischemia caused by spontaneous TOH in a virgin abdomen. This case makes a significant contribution to the literature because it presents a rare clinical scenario that can provide a diagnostic framework for avoiding misidentification and details a novel and successful laparoscopic intervention for which standardized protocols are currently lacking.

## 2. Case Presentation

A 71‐year‐old woman with a negative surgical history but a medical history of diabetes mellitus, hypertension, and dyslipidemia presented to the emergency department with a 12‐h history of sudden‐onset severe periumbilical abdominal pain, nausea, and bilious vomiting. The last bowel motion occurred 2 days prior to presentation. Upon examination, the patient had tachycardia (102 bpm) and hypotension (90/60 mmHg). Abdominal examination revealed mild tenderness throughout the abdomen with guarding. A digital rectal examination revealed an empty ampulla.

**Figure 1:**
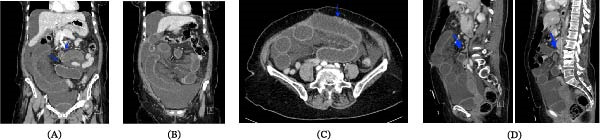
Computed tomography images. (A) Coronal image showing a dilated small bowel with two transition zones, suggesting a closed‐loop obstruction due to a transmesenteric internal hernia. (B) Coronal image of the abdomen showing dilated small bowel segments with mesenteric edema and fluid. A moderate ascites volume is also visible. (C) Axial image through the lower abdomen showing a dilated small bowel segment with mural thickening and decreased mural enhancement, suggesting bowel ischemia. (D) Sagittal images of the abdomen showing dilated small bowel segments with two transition zones (arrows) as well as mesenteric edema and free fluid.

Laboratory findings showed leukocytosis (13,000 white blood cells/µL) and compensated metabolic acidosis, with an elevated serum lactate level (4.1 mmol/L), suggesting bowel ischemia. Contrast‐enhanced computed tomography (CT) revealed dilated small bowel loops with two transition points, consistent with a closed‐loop obstruction, possibly due to a transmesenteric internal hernia (Figure 1). Findings of bowel wall thickening and decreased enhancement raised concerns about ischemia [5, 6].

**Figure 2:**
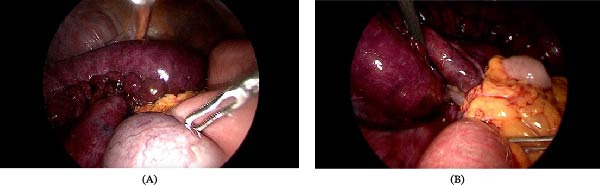
Intraoperative findings. (A) Dilated small bowel with an ischemic loop and (B) herniation of the small bowel with the engorged mesentery through a defect in the greater omentum.

The patient underwent laparoscopic exploration. Intraoperatively, an ischemic loop of the proximal ileum of ~1.5 m was identified (Figure 2A). A TOH was found to be the cause of the obstruction, with a segment of the small bowel herniating through a defect in the greater omentum (Figure 2B). The compressed mesentery was released by dividing the omentum. After 15 min of observation, the bowel color, peristalsis, and mesenteric pulse did not improve, necessitating laparoscopic resection of the 1.5‐m ischemic segment and anastomosis with closure of the new mesenteric defect. After resection, the total measured length of the remaining bowel was ~4 m. The resected bowel was then exteriorized through one of the incisions, which was extended to 3 cm. The postoperative course was uneventful, and the patient was discharged on postoperative day 5. During the 1‐year postoperative follow‐up period, there were no signs of short bowel syndrome or any significant nutritional side effects.

## 3. Discussion

Internal hernias occur when the viscera protrude through a peritoneal or mesenteric defect while remaining within the abdominal cavity. Internal hernias are classified based on the anatomical herniation site as paraduodenal (53%), paracecal (13%), foramen of Winslow hernia (8%), transmesocolic (8%), intersigmoid hernia (6%), and TOH (1%–4%). TOHs are the rarest subtype and are characterized by the abdominal viscera, commonly the small bowel, protruding through a defect in the greater or lesser omentum [7]. TOHs occur spontaneously or secondary to iatrogenic, inflammatory, or traumatic causes [3, 8, 9]. Their incidence is higher in older patients and is related to senile atrophy. Recently, the incidence of iatrogenic TOH has increased following an increase in the number of Roux‐en‐Y gastric bypasses performed for bariatric reasons. It has a high risk of strangulation owing to its narrow hernial orifice, leading to compromised mesenteric circulation [2, 4].

Yamaguchi categorized TOHs into three types based on the herniated bowel pathway: Type A (peritoneal cavity → greater omentum → peritoneal cavity), Type B (peritoneal cavity → omental bursa → peritoneal cavity), and Type C (peritoneal cavity → omental bursa), which is further subdivided into types C0, C1, and C2 based on the herniation pathway [7, 10]. In the present case, the hernia corresponded to Type A as the small bowel herniated through a defect in the greater omentum into the peritoneal cavity. Tsuchida et al. [11] analyzed the data of 188 patients diagnosed with TOH and found that Type A was more common than Type C in older adult patients (107 vs. 70 patients); no Type B TOHs were detected. Furthermore, 38.8% of the patients (73 of 188) underwent bowel resection because of irreversible ischemic changes. Most of these patients were older, had a narrow hernia orifice, and had a long herniated segment of the bowel. These findings may reflect the difficulty in the early diagnosis of the disease, resulting in delayed surgical intervention [2]. Patients who present with TOH‐related bowel obstruction tend to have classical abdominal symptoms, such as abdominal pain, distention, nausea, vomiting, and constipation. As TOHs do not have a hernia sac and the orifice size is small, they tend to be strangulated more often than other types of internal hernias. Consequently, the postoperative mortality rate is as high as 30%, which highlights the importance of early diagnosis and management [7]. Nonetheless, an early preoperative diagnosis is challenging because of its non‐specific presentation. CT imaging plays a critical role, with findings such as the “beak sign” and “whirl sign” of mesenteric vessels, as well as dilated bowel loops in the lesser sac or over the transverse colon, suggesting TOHs [6, 9]. In TOHs, the neck of the hernia can be seen traversing the omental fat. However, most cases are definitively diagnosed intraoperatively during surgical exploration [1, 2].

Surgical intervention is the gold standard of treatment. The herniated bowel must be reduced, the defect in the omentum must be closed to prevent recurrence, and in cases of irreversible ischemic changes, bowel resection must be performed [5, 7]. As demonstrated in this case, laparoscopic surgery is a feasible approach with expertise, proper equipment, and intensive care support. The available literature strongly supports the use of a laparoscopic approach for the management of strangulated bowel obstruction in a carefully selected patient population. As TOH is a very rare type of internal hernia, few studies have described the laparoscopic and open approaches in cases of strangulation. Therefore, we referred cases of strangulated groin hernias. In this case, the laparoscopic approach was chosen because of the potential benefits of minimally invasive surgery, such as better outcomes in healing, pain, mobilization, and cosmetic results without compromising patient safety. In my practice, I begin the procedure with an exploratory laparoscopy if there are no signs of generalized peritonitis. If I deem that the condition can be managed in a minimally invasive manner, I proceed. Otherwise, I switch to the open approach.

A systematic review and meta‐analysis of 10 studies, involving 1250 patients, comparing laparoscopic and open repair for strangulated inguinal hernia revealed that laparoscopic repair for strangulated inguinal hernia was associated with shorter hospital stays and fewer wound infections but required longer operative time than open repair. Both techniques are comparable in terms of recurrence and mortality rates [12]. Another systematic review of eight studies with 316 patients aimed to evaluate the feasibility and safety of transabdominal preperitoneal (TAPP) repair for incarcerated and strangulated groin hernias. The study showed that laparoscopy is a transformative approach and that TAPP repair is a feasible, safe, and effective technique for the emergent repair of strangulated groin hernias [13]. Furthermore, Farrell et al. [14] conducted a review of 34 studies to provide evidence‐based guidelines for managing inguinal hernias requiring urgent surgical intervention. Findings from the study showed that laparoscopic repairs led to lower recurrence rates and shorter length of hospital stay than open repairs; hence, the laparoscopic approach was recommended over the open approach for this population. A prospective clinical trial that analyzed the outcomes between the open and laparoscopic approaches for incarcerated and strangulated hernias showed that the laparoscopic approach for acutely incarcerated/strangulated groin and obturator hernias is effective, safe, and feasible [15]. Similarly, a retrospective study analyzed and compared the results of 188 patients managed with an emergency laparoscopic or open repair for strangulated groin hernias. The results showed that laparoscopic repair for strangulated groin hernias is feasible and appears to be associated with lower morbidity relative to open repair [16]. Numerous case reports on laparoscopic repair of external and more common types of internal hernia (compared to the type reported here) also strongly support the use of a laparoscopic approach for managing strangulated bowel obstruction in carefully selected patients. Laparoscopic surgery positively affects patient recovery, reducing postoperative pain and enhancing mobilization, with a lower incidence of incisional hernia and wound infection.

TOHs are a rare, life‐threatening cause of small bowel obstruction with a high risk of ischemia because of the narrow hernia orifice and herniation of the long segment of the bowel. Early recognition using contrast‐enhanced CT and prompt surgical exploration can lead to favorable outcomes. Surgeons should be aware of this condition, particularly in older adult patients presenting with acute small bowel obstruction [2, 6, 7].

When performed by an experienced surgeon, laparoscopy is a feasible approach that can elicit significant advantages over traditional open repair, including lower morbidity, shorter hospital stays, and faster recovery, without compromising recurrence and mortality outcomes. While challenges related to technical difficulty and anesthesia requirements persist, the benefits of enhanced visualization and a minimally invasive approach make it a highly valuable tool in the armamentarium of emergency general surgery. As surgical expertise and technology continue to advance, the role of laparoscopy in the treatment of this urgent condition is likely to expand further.

## Funding

No funding was received for this manuscript.

## Ethics Statement

No written consent has been obtained from the patients as there are no patient identifiable data included in this case report.

## Conflicts of Interest

The author declares no conflicts of interest.

## Data Availability

The data that support the findings of this study are available from the corresponding author on reasonable request.
